# Unusually complex phase of dense nitrogen at extreme conditions

**DOI:** 10.1038/s41467-018-07074-4

**Published:** 2018-11-09

**Authors:** Robin Turnbull, Michael Hanfland, Jack Binns, Miguel Martinez-Canales, Mungo Frost, Miriam Marqués, Ross T. Howie, Eugene Gregoryanz

**Affiliations:** 1 Centre for Science at Extreme Conditions and School of Physics and Astronomy, University of Edinburgh, Edinburgh, UK; 2 European Synchrotron Radiation Facility, Grenoble, France; 3 Center for High Pressure Science & Technology Advanced Research, Shanghai, China; 4 SLAC National Accelerator Laboratory, Menlo Park, CA USA; 5 Key Laboratory of Materials Physics, Institute of Solid State Physics, Chinese Academy of Sciences, Hefei, China

## Abstract

Nitrogen exhibits an exceptional polymorphism under extreme conditions, making it unique amongst the elemental diatomics and a valuable testing system for experiment-theory comparison. Despite attracting considerable attention, the structures of many high-pressure nitrogen phases still require unambiguous determination. Here, we report the structure of the elusive high-pressure high-temperature polymorph *ι*–N_2_ at 56 GPa and ambient temperature, determined by single crystal X-ray diffraction, and investigate its properties using ab initio simulations. We find that *ι*–N_2_ is characterised by an extraordinarily large unit cell containing 48 N_2_ molecules. Geometry optimisation favours the experimentally determined structure and density functional theory calculations find *ι*–N_2_ to have the lowest enthalpy of the molecular nitrogen polymorphs that exist between 30 and 60 GPa. The results demonstrate that very complex structures, similar to those previously only observed in metallic elements, can become energetically favourable in molecular systems at extreme pressures and temperatures.

## Abstract (web summary)

Nitrogen has a complex phase diagram with rich polymorphism, which is challenging to characterize due to the extreme conditions and uncertain stability ranges needed to do so. Here the authors resolve one of the most elusive phases of this model system, reporting a crystalline structure with unusual complexity.

## Introduction

Extreme-conditions research has uncovered a new regime of complex structures in elemental materials which, counter-intuitively, deviate from simple close packing with the application of pressure. This pressure-induced complexity has previously been exemplified by the structural diversity observed in light alkali and alkaline-earth metals^1–3^. In contrast, such complex, large volume structures have neither been observed, nor have they been predicted, in elemental simple-molecular systems. The nitrogen molecule is used to explore high-pressure phenomena in experimental and theoretical extreme-conditions research because its significant polymorphism makes it unique amongst diatomic elements. Since the first cryogenic experiments of the early 20th century, 15 unique phases of solid nitrogen have been reported over a wide range of pressures and temperatures, including: 12 molecular phases^4–12^, two non-molecular phases^13,14^ and an amorphous state^15^, as well as numerous predicted structures^16–20^. The majority of the phases were detected through spectroscopic techniques, which, although useful for providing structural ‘fingerprints’, do not reveal the underlying crystal structures. Consequently, the structures of many of the nitrogen phases remain unknown despite experimental efforts, due primarily to challenges associated with producing and analysing single-crystal samples in the geometrically constrained environment of diamond anvil cells (DACs).

**Fig. 1:**
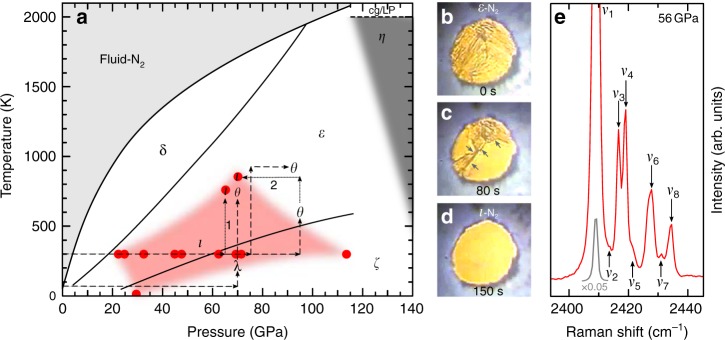
The nitrogen phase and reaction diagram. **a** The reported *P*–*T* paths to the high-temperature molecular phases *ι*–N_2_ and *θ*–N_2_ are shown with dotted and dashed arrows respectively. The red shaded region approximates the known stability field of *ι*–N_2_ based on a combination of our Raman measurements and those of ref.^12^. Path 1: Isobaric heating of *ε*–N_2_ to 750 K at 65 GPa, as performed in this study. Path 2: Isothermal decompression of *θ*–N_2_ to 69 GPa at 850 K. *P*–*T* paths and data points are taken from refs.^11,12,21,22^ which identify the phases through Raman spectroscopy. Black phase-boundaries are based on refs.^21–23^. Phases *α*, *β*, *γ*, δ^*^, *ζ*^'^, *κ* and *λ*–N_2_ are omitted for clarity. **b**–**d** Micrographs of the visual changes across the *ε*–N_2_ → *ι*–N_2_ phase transition. The sample is approximately 60 μm in diameter and 15 μm thick. The time from the onset of the phase-transition is shown on each frame. The arrows in **c** indicate the progression of the *ε*–N_2_ → *ι*–N_2_ phase boundary. **e** Vibrational Raman spectrum of *ι*–N_2_ once recovered to ambient temperature. The inset spectrum (light grey) shows *ν*_1_ scaled by a factor of 0.05 to display it fully

The pressure-temperature (*P*–*T*) phase and reaction diagram of nitrogen (illustrated in Fig. 1a) has been a focal point in high-pressure research for the past 15 years since the discovery of the high-temperature high-pressure phases^12^ known as *ι*–N_2_ and *θ*–N_2_. The phase which nitrogen adopts strongly depends on the *P*–*T* history of the system, and both the *ι*–N_2_ and *θ*–N_2_ phases are recoverable to ambient temperature and much lower pressures than those required for their synthesis^12^ (shown in Fig. 1a). The structures *ι*–N_2_ and *θ*–N_2_ have remained unknown since their discovery. The *ι*–N_2_ phase has proved particularly elusive, with only the original synthesis study providing experimental data^12^ which was limited to Raman and infra-red spectroscopy. Subsequently, one work claimed the production of *ι*–N_2_ after temperature-quenching fluid–N_2_ at high-pressure but provided no supporting evidence^21^. Other studies have been unable to observe *ι*–N_2_ despite replicating the correct *P–T* conditions^22^, instead reporting recrystallization of fluid–N_2_ into the conventional *δ*–N_2_ and *ε*–N_2_^23^, thereby casting ambiguity over the *P*–*T* path and conditions at which *ι*–N_2_ can be synthesised.

## Results

### *ι*–N_2_ synthesis and Raman spectra

In this study we have unequivocally synthesised a single crystal of *ι*–N_2_ in a resistively heated DAC from *ε*–N_2_ at 65 GPa and 750 K. (See Methods section for further details). The *ε*–N_2_ → *ι*–N_2_ transition was initially identified by Raman spectroscopy and visual observation, as shown in Fig. 1b–e. The transition is characterised by the loss of the *ε*–N_2_ *ν*_1_ vibrational mode, as shown in the Raman spectra across the synthesis *P*–*T* path in Supplementary Figure 1. A video of the phase transition can be seen in Supplementary Movie 1. Typically, *ε*–N_2_ has a birefringent appearance which became uniformly smooth on entering the *ι*–N_2_ phase. Phase boundaries between *ε*–N_2_ and *ι*–N_2_ were clearly observed moving across the sample chamber (also indicated by arrows in Fig. 1c) and the transition progressed to completion over several minutes, indicating a kinetically slow phase transition which requires the sustained elevated temperatures generated by resistive heating techniques. This observation may explain why previous studies, which utilised laser heating, have struggled to produce the *ι*–N_2_ phase at the same *P*–*T* conditions as the technique suffers from transient and localised peak-temperatures within the sample^24^. Additionally, Raman spectra of the *ι*–N_2_ → *ε*–N_2_ back-transformation on isothermal decompression at ambient temperature (Supplementary Figure 2) show the re-emergence of the *ε*–N_2_ *ν*_1_ vibrational mode between 20–25 GPa, in agreement with ref.^12^.

After recovery to ambient temperature, high resolution Raman spectra were acquired to confirm the synthesis of *ι*–N_2_ (Fig. 1e). In total eight vibrational modes were resolved, all of which originate from molecular N_2_ centres. The lowest frequency vibrational mode, *ν*_1_ (2409 cm^−1^ at 56 GPa), is the most intense by approximately an order of magnitude. The additional higher frequency vibrational modes (*ν*_2_–*ν*_8_) are all within 30 cm^−1^ of *ν*_1_, describing an unusually complex vibrational spectrum for an elemental molecular system. The lattice modes of *ι*–N_2_, shown in Supplementary Figure 3, appear sharp and well-defined indicating complete orientational ordering which is consistent with the *ι*–N_2_ structure described below.

### X-ray crystallography and structure determination

**Fig. 2:**
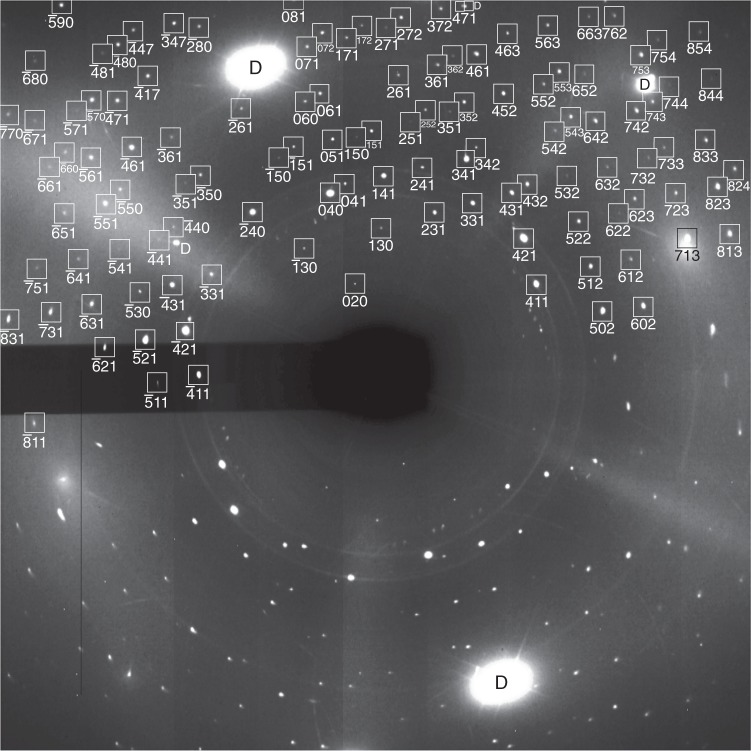
Single crystal X-ray diffraction pattern of *ι–*N_2_ at 56 GPa. The data were collected over a 56° scan range upon quenching the sample to ambient temperature. Squares mark nitrogen reflections with the given *hkl* indices. Diamond reflections are labelled with the letter *D*. Indices are not shown on the lower half of the image plate to clearly display the quality of the raw data

**Fig. 3:**
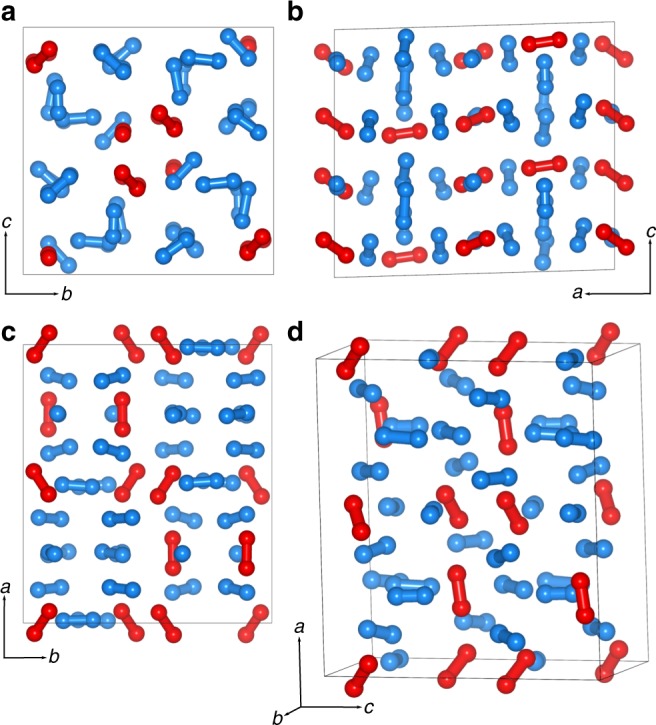
The refined crystal structure of *ι*–N_2_. **a**–**c** Projections along the *a*, *b* and *c* axes respectively. **d** A perspective projection of the unit cell. Layered N_2_ molecules are shown in blue and oriented molecules are shown in red. Supplementary crystallographic data for the *ι*–N_2_ structure can be obtained free of charge from The Cambridge Crystallographic Data Centre, under deposition number CCDC 1869044

The crystal structure of *ι*–N_2_ was determined by single-crystal synchrotron X-ray diffraction (XRD) at 56 GPa and ambient temperature. The *ι*–N_2_ crystal was of extremely high quality with little strain as evidenced by the very well-defined reflections shown in Fig. 2. The data were integrated to a resolution limit of 0.6 Å with an excellent low *R*_int_ = 0.0342 for a total of 1276 (641 unique) reflections. Details of the XRD data collection and refinement are given in Supplementary Table 1 and atomic positions in Supplementary Table 2. The reflections were indexed to a primitive monoclinic lattice with unit-cell dimensions of: *a* = 9.899(2), *b* = 8.863(2), *c* = 8.726(2) Å, *β* = 91.64(3)° and *V* = 765.2(3) Å^3^ at 56 GPa. Systematic absence analysis unambiguously indicates the space group is *P*2_1_/*c* and previously proposed structures can be discarded^25^. Projections of the refined *ι*–N_2_ crystal structure are shown in Fig. 3. The unit cell is by far the largest observed for any elemental diatomic phase, containing 12 crystallographically unique N_2_ molecules, giving a total of 48 N_2_ molecules per unit cell. The refined N–N bond lengths are all typical for molecular nitrogen, and the estimated standard deviations on the bond lengths and atomic positions (given in Supplementary Table 3) are also low, providing further confidence in the model. For comparison, the next largest nitrogen unit cells belong to *ε*–N_2_ and δ^*^–N_2_, which contain 24 and 16 molecules respectively^6,10^. The *ι*–N_2_ unit cell is nearly tetragonal about the *a* axis, with *b* and *c* differing by 0.127(2) Å. Of the 48 molecules in the unit cell, 12 are oriented primarily along the *a* axis (shown in red) and the remaining 36 are layered in the *bc* plane (shown in blue in Fig. 3). Each unit cell contains eight layers, half of which contain axial molecules, with interlayer distances varying between 1.1720(2) and 1.2639(3) Å depending upon the orientation of the axial molecules in the containing layer.

### Density functional theory (DFT) calculations

**Fig. 4:**
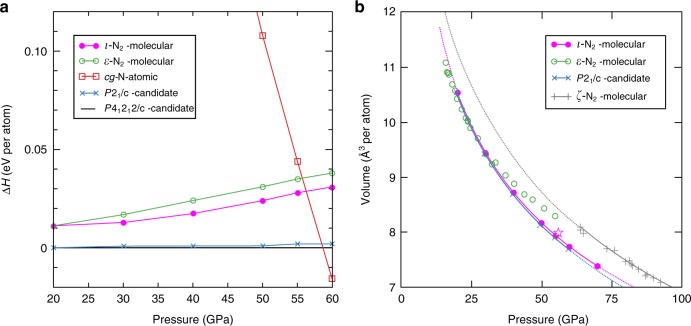
The calculated enthalpy and volume per atom for the *ι*–N_2_ structure. **a** Calculated PBE enthalpy differences with respect to the *P*4_1_2_1_2-candidate structure of ref.^18^. The computed enthalpy of *ι*–N_2_ is more favourable than *ε*–N_2_ above 20 GPa. Polymeric *cg*–N becomes favourable at 58 GPa, in agreement with previous DFT estimates. **b** Calculated volume per atom of *ι*–N_2_ and the *P*2_1_/*c*-candidate structure of ref.^18^ plotted with experimental data for *ε*–N_2_ and *ζ*–N_2_. The *ι*–N_2_ volumes are ~1% larger than the *P*2_1_/*c*-candidate of ref.^18^. The star shows the experimentally determined *ι*–N_2_ volume per atom at 56 GPa and ambient temperature. The experimental data for *ε*–N_2_ and *ζ*–N_2_ are reproduced from ref.^9^ and references therein

The refined *ι*–N_2_ structure was analysed using DFT within the standard PBE functional approximation^26^ and the results found *ι*–N_2_ to be energetically favourable. In geometry optimisation calculations the atomic positions quickly converged to positions, on average, less than 0.005 Å away from the experimentally refined ones, again underlining confidence in the proposed structure. The enthalpy of *ι*–N_2_ was also found to be very competitive (Fig. 4a) and, at the PBE level, better than that of *ε*–N_2_ beyond 20 GPa, despite *ι*–N_2_ forming at high temperature, suggesting that entropy plays a role in its stability. *ι*–N_2_ was also compared with theoretical low enthalpy candidate high-pressure nitrogen structures proposed by ref.^18^ (shown in Fig. 4) which were found to be lower in enthalpy than *ι*–N_2_ by between 10 and 25 meV/atom over the pressure region of interest. However none of the proposed structures of ref.^18^ have been observed experimentally. (Note that although the ‘*P*2_1_/*c*-candidate’ of ref.^18^ shares its space group with *ι*–N_2_, it contains only two molecules). The calculated *ι*–N_2_ volume per atom shows a volume decrease of approximately 2.5% compared to *ε*–N_2_ at ambient temperature and 56 GPa (Fig. 4b) which is consistent with a spontaneous high-pressure phase transition such as the one observed here.

The calculated Raman spectrum of *ι*–N_2_ (Supplementary Figure 4) reproduces the high frequency vibrational structure, with eight active modes, of which the lowest frequency mode is at least an order of magnitude more intense than the others. The calculated intensities of the low frequency modes (Supplementary Figure 5) are two orders of magnitude lower than those of the most intense vibron, in close agreement with experiment. Additionally, ab initio random structure searches (AIRSS) were performed using all available experimental *ι*–N_2_ constraints, such as the lattice parameters, space group (subgroups were allowed), and number of molecules per cell. The details of the searches can be found in the Methods section below. The experimentally observed *ι*–N_2_ structure was yielded twice, and, of >3100 structures generated, none had a lower enthalpy than *ι*–N_2_, with the next best being a purely molecular structure at 26 meV/molecule greater than *ι*–N_2_. Such large systems are not typically attempted with contemporary AIRSS techniques due to computational constraints, and it is certainly noteworthy that *ι*–N_2_ was found despite the large unit cell and weak intermolecular interactions. Unconstrained and partially constrained searches favour polymerization at these pressures, resulting in mixed molecular/non-molecular phases similar to those predicted previously^20,27^. It is therefore extremely unlikely that the *ι*–N_2_ structure would have been found without the experimental constraints, thus highlighting the importance of close collaboration between theory and experiment in the search for high-pressure structures.

## Discussion

Our study demonstrates that the appearance of complex structures under pressure is not limited only to metallic elements^1–3^ and raises the intriguing question of whether unusual phases could be expected in other molecular systems at extreme conditions. The pressure-induced complexity in many metallic elements has been attributed to *s-p* or *s-d* electronic transfer^28,29^ which is clearly not an applicable stability mechanism in the case of the molecular and insulating *ι*–N_2_ phase. Therefore, there must be other mechanisms in highly condensed nitrogen which make these very complex configurations favourable. In the ‘disorder-order’ transition from *δ*–N_2_ to *ε*–N_2_, the N_2_ molecules forego the entropy of their rotational disorder, in favour of occupying less volume, by ordering in certain molecular orientations^10^. In the transition from *ε*–N_2_ to *ι*–N_2_ however, both phases are characterised by rotationally ordered N_2_ molecules. So, what drives the ‘order-order’ transition? In this case the transition is thermodynamically favourable because the volume per atom in the unit cell decreases (as seen in Fig. 4b), whilst the configurational entropy increases due to the larger number of molecules in the unit cell. The *ε*–N_2_ to *ι*–N_2_ transition is additionally characterised by a rearrangement of the N_2_ molecules into layers (as seen in Fig. 3) which may facilitate vibrational resonance coupling, particularly because molecules between layers take a mutually perpendicular orientation^30^, which could play an important role in kinetic favourability. Our DFT calculations find *ι*–N_2_ to have the lowest enthalpy of the molecular nitrogen polymorphs for which the structures are known between 30 and 60 GPa, which will have implications for the nitrogen phase diagram. Although some pioneering structural searches on nitrogen^31^ have been later confirmed by experiment^13^, these structures were elegantly simple and identified by comparison of powder XRD data with calculated patterns. Our study of *ι*–N_2_ shows that in systems with unforeseen complexity, interesting structures could be potentially overlooked without the direction offered by experiment, and that at extreme conditions unusually complex structures may be more energetically favourable in molecular systems. We hope that our results will prompt further investigations into why such complex structures should appear at high pressures in elemental diatomic solids.

## Methods

### Sample preparation

Research-grade nitrogen (>99.9995% purity, obtained from BOC) was condensed at 77 K and loaded into symmetric DACs equipped with built-in graphite resistive-heaters. Wide opening seats with Boehler-Almax diamonds were used to allow for maximal scattering angle in XRD experiments. Culet sizes were 200 μm and rhenium gaskets were pre-indented to 20 μm prior to loading. Raman spectroscopy was conducted using the 514 nm emission line of an Ar^+^ ion laser. Pressure was controlled via a gas-membrane and pressure was determined using the diamond edge scale^32^. The samples were recovered to ambient-temperature and transported by applying the load of the gas-membrane onto the DAC screws.

### XRD and structural refinement procedure

Single-crystal XRD data used for refinement were collected at the ID15B beamline at ESRF (Grenoble, France) using a monochromatic beam *λ* = 0.411 Å focused to a spot size of 10 × 10 μm. Data were recorded on a MAR555 flat panel detector. The sample was rotated about *ω* over a range of 60° in 0.5° increments. The image-plate shown in Fig. 2 was acquired at the Extreme Conditions Beamline (P02.2) at PETRA III (Hamburg, Germany) using a monochromatic beam *λ* = 0.2889 Å focused to a spot size of 4 × 4 μm. Data were recorded on a PerkinElmer XRD 1621 detector. Data were indexed and integrated with CrysalisPro^33^. A total of 1276 reflections were indexed to a monoclinic lattice with unit-cell dimensions *a* = 9.899(2), *b* = 8.863(2), *c* = 8.726(2) Å, *β* = 91.64(3)°, *V* = 765.2(3) Å^3^ at 56 GPa. Systematic absence analysis clearly indicated space group *P*2_1_/*c*. Data were integrated to a resolution of 0.6 Å with a merged *R*_int_ = 0.0342 for 641 unique reflections, a completeness of 18%. The crystal was oriented with the c* axis approximately perpendicular to the diamond culets resulting in a very low data coverage along c* (−15 ≤ *h* ≤ 15, − 14 ≤ *k* ≤ 13, − 5 ≤ *l* ≤ 4). The structure was solved by dual-space methods using SHELXT and refinement of the crystal structure was carried out against |*F*^2^| with the SHELXL refinement package^34,35^. Refinement was limited to an isotropic treatment for all nitrogen atoms, an anisotropic treatment led to refinement instabilities. Rigid bond restraints were applied to all molecular bonds with default estimated standard deviations of 0.004 Å^2^. Intramolecular bond distance restraints do not improve the fit to the data.

### DFT calculations

DFT calculations of energetics and vibrational spectra were performed with CASTEP^36^ 18.1. Energetics were computed using the default CASTEP 18 ultrasoft pseudopotential, a 700 eV energy cutoff and a k-point grid spacing better than 0.04 Å^−1^. In order to estimate the effect of neglected non-local van der Waals interactions, enthalpies were recalculated including Tkatchenko-Scheffler corrections^37^. The effect is negligible, other than lowering the pressure of the transition to the polymeric *cubic gauche* (*cg*–N) phase. Raman spectra were computed using the DFPT + Finite differences method, using the default CASTEP 18 NC pseudopotentials with a 1250 eV energy cutoff, and a similarly dense k-point grid.

### Structure searches

Ab initio evolutionary structural searches for the *ι*–N_2_ structure were performed with the USPEX^38^ code and VASP^39,40^. The initial search space with 1000 structures consisted of atoms and N_2_ molecular units. The experimentally proposed *P*2_1_/*c* symmetry was imposed on some of the searches, but subgroups of lower symmetry such as *P*1̄, *Pc* and *P*2_1_ were also considered. Initial searches with 104 atoms in the unit cell lead to structures ≈0.052 eV/atom higher in enthalpy than those with 96 atoms.

A random structure search was performed with AIRSS^41^ and CASTEP 18.1, on the experimental cell with 96 atoms. In total, >3100 structures were produced. The most favourable searches were those done enforcing initial N_2_ molecules and symmetry, generating structures belonging to the *P*2_1_/*c* space group, as well as its subgroups *P*2_1_ and *Pc*. We were able to reproduce the experimentally refined cell twice, and this was the lowest enthalpy structure found under these considerations.

## Electronic supplementary material

Supplementary Information

Description of Additional Supplementary Files

Supplementary Movie 1

## Data Availability

Supplementary crystallographic data for the *ι*–N_2_ structure can be obtained free of charge from The Cambridge Crystallographic Data Centre, under deposition number CCDC 1869044, via www.ccdc.cam.ac.uk/structures. The relevant *ι*–N_2_ DFT enthalpies, computed Raman spectra and AIRSS calculation data are accessible from the Edinburgh DataShare repository via 10.7488/ds/2449. All relevant data are available from the corresponding author upon reasonable request.
