# Supplementary material for: Unusually complex phase of dense nitrogen at extreme conditions
Source: Nat Commun. 2018 Nov 9;9:4717. doi: 10.1038/s41467-018-07074-4 (PMC6226474; doi:10.1038/s41467-018-07074-4)
Supplement: Supplementary file 1 — Supplementary Information [file 41467_2018_7074_MOESM1_ESM.pdf]

Supplementary Information for Unusually Complex Phase of Dense Nitrogen at Extreme Conditions

Robin Turnbull[1], Michael Hanfland[2], Jack Binns[3], Miguel Martinez-Canales[1], Mungo Frost[1,4], Miriam Marqués[1], Ross T. Howie[3] and Eugene Gregoryanz[5,3,1,*]

[1] Centre for Science at Extreme Conditions and School of Physics and Astronomy, University of Edinburgh, Edinburgh, UK.

[2] European Synchrotron Radiation Facility, Grenoble, France.

[3] Center for High Pressure Science & Technology Advanced Research, Shanghai, China.

[4] SLAC National Accelerator Laboratory, Menlo Park, California, USA.

[5] Key Laboratory of Materials Physics, Institute of Solid State Physics, Chinese Academy of Sciences, Hefei, China.

*e-mail: eugene@issp.ac.cn

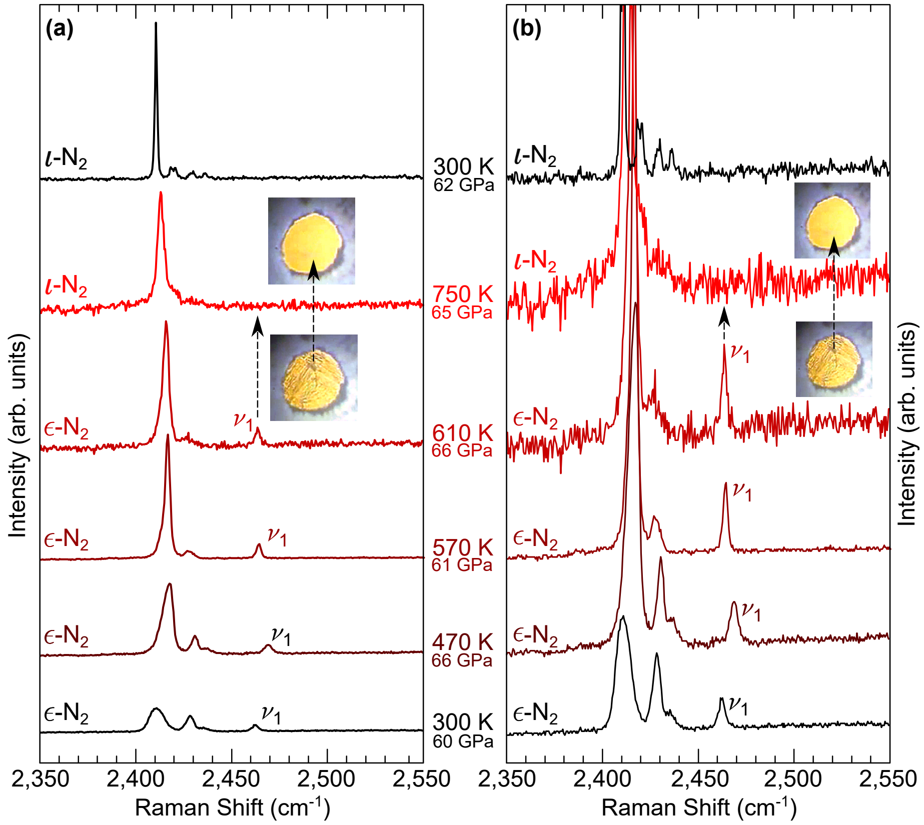

Supplementary Figure 1 | Raman spectra of the $\epsilon-N_2$ to $\iota-N_2$ transition along the isobaric heating synthesis *P-T* path. The data in **(a)** and **(b)** are identical except for an intensity scaling factor to emphasise the smaller vibrational modes. The phase transition is characterised by the disappearance of the $\epsilon-N_2$ ν_1 vibrational mode at high temperature. (For further information on the vibrational spectra of $\epsilon-N_2$ see ref. 1.) The transition was also observed visually as shown in the inset images and in Supplementary Movie 1. Raman spectra were not collected during the quench to ambient temperature because recovery of the $\iota-N_2$ single crystal was of paramount importance in order to collect single crystal XRD data. Experimentally this technique is very challenging and involves continuously monitoring the sample pressure whilst simultaneously counterbalancing the temperatre induced pressure changes by adjusting the applied load on the DAC. DAC pressure can increase very quickly on cooling, potentially shattering the brittle $\iota-N_2$ crystal which was crucial for determining the atomic positions.

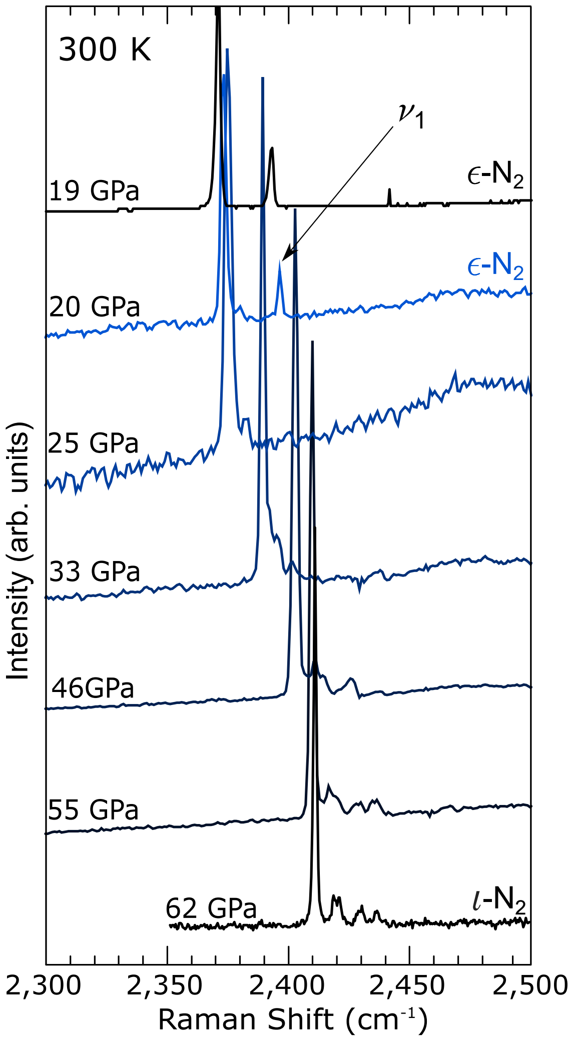

Supplementary Figure 2 | Raman spectra of the $\iota-N_2$ to $\epsilon-N_2$ back-transformation on isothermal decompression at ambient temperature. The phase transition is characterised by the appearance of the $\epsilon-N_2$ ν_1 vibrational mode (i.e. the same mode which disappears in the forward-transformation on isobaric heating in Supplementary Figure 1).

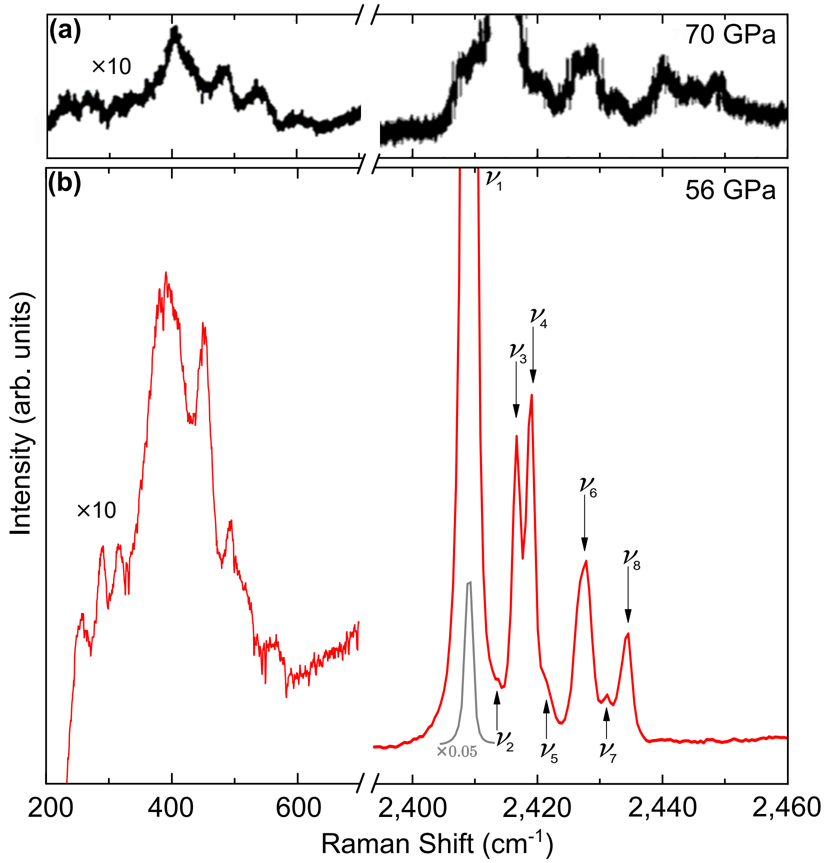

Supplementary Figure 3 | Raman spectra of ι−N_2 at ambient-temperature. **(a)** The original spectra taken from ref. 2 at 70 GPa. **(b)** The new spectra of the current work at 56 GPa confirming the synthesis of ι−N_2. The higher shifts observed in **(a)** are due to the difference in sample pressure. The intensity of the low-frequency modes has been multiplied by a factor of 10 for clarity. The inset spectrum (light grey) in **(b)** shows ν_1 scaled by a factor of 0.05 to display it fully. The peak in the left shoulder of most intense vibrational mode of ι−N_2 in **(a)** is probably due to the presence of residual ζ−N_2 (which was used as a starting material) in the polycrystalline sample of ref. 2. In the ι−N_2 single crystal sample of the current work no contamination was observed in XRD and the Raman shoulder was not present. Additionally, the phase transition from ϵ−N_2 at the same *P*-*T* conditions as originally reported in ref. 2 which further adds confidence to the synthesis of ι−N_2.

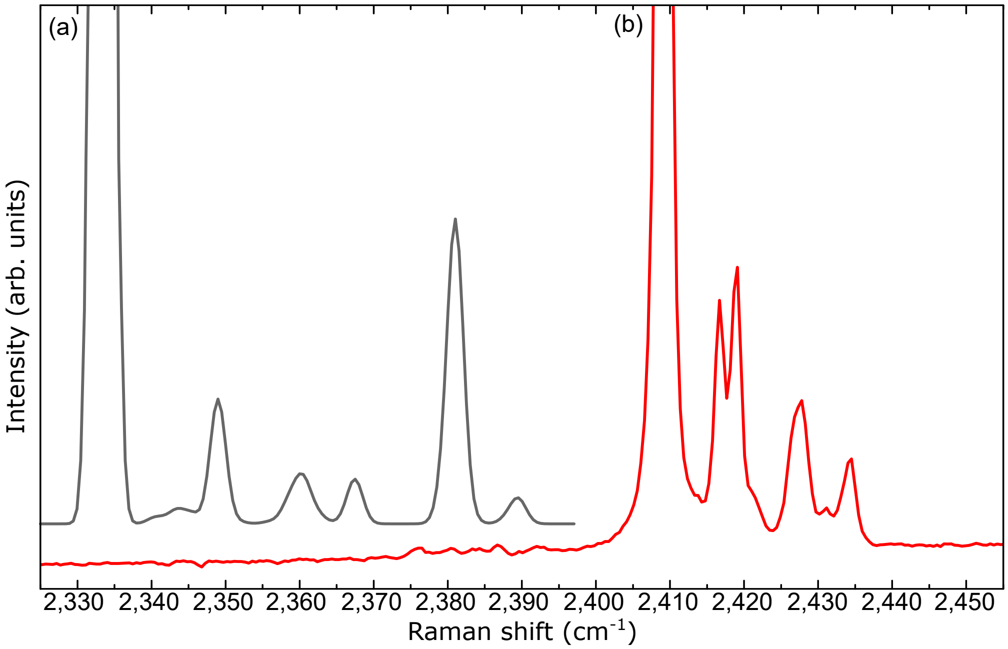

Supplementary Figure 4 | Vibrational Raman spectra of $\iota-N_2$ (a) Calculated Raman vibrational spectrum of $\iota-N_2$ at 55 GPa using the experimentally refined structure. **(b)** Experimental Raman vibrational spectrum of $\iota-N_2$ at 56 GPa. The calculated vibrational spectrum reproduces the high frequency vibron structure, with eight active modes, of which the lowest frequency mode is at least an order of magnitude more intense than the others. It is important to note that the figure spans a very narrow region of approximately 130 cm^{-1} and the agreement between theory and experiment to within 70 to 80 cm^{-1} is very good. Owing to the large number of modes, the calculated low frequency spectrum ($<$ 500 cm^{-1}) is complicated (see Supplementary Figure 5), and the frequencies are in less quantitative agreement with experiment. However, the intensities are two orders of magnitude lower than those of the most intense vibron, in good agreement with experiment. One of the librons is weakly unstable ($\nu = 31i\text{cm}^{-1}$), possibly due to a temperature-induced symmetrisation.

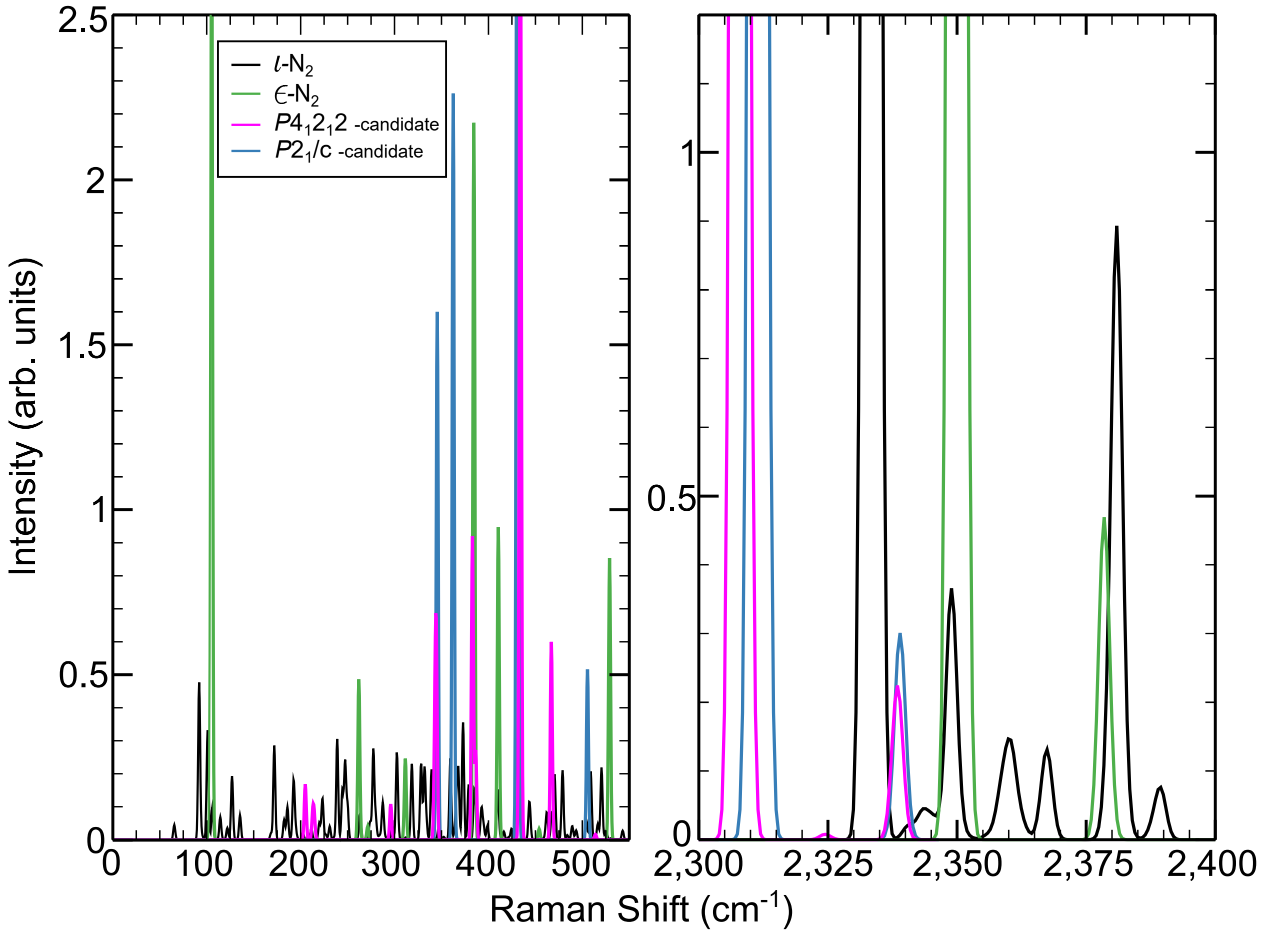

Supplementary Figure 5 | Computed Raman spectra of the candidate high-pressure nitrogen structures proposed by ref. 3. Each spectrum has been normalised so the strongest vibron has an intensity of 10 arbitrary units. The computed Raman spectra of the low-energy molecular structures proposed by ref. 3 ('$P4_12_12$-candidate' and '$P2_1/c$-candidate') show that only $\iota-N_2$ is in qualitative and quantitative agreement with the experimental data.

Crystal data	
Space group	$P2_1/c$
a (Å)	9.899(2)
b (Å)	8.863(2)
c (Å)	8.726(2)
β (°)	91.64(3)
V (Å^3)	765.2(3)
Data collection	
Number of measured reflections	1276
Number of unique reflections	641
Number of observed reflections [$\|F_{obs}\| > 2\sigma$]	390
R_{int}	0.034
Refinement	
$R[F^2 > 2\sigma(F^2)]$	0.123
Number of parameters	97
Number of restraints	0
$\Delta\rho_{max}$, $\Delta\rho_{min}$ (e Å^{-3})	$0.445, -0.439$

Supplementary Table 1 | Data collection and refinement details for $\iota-N_2$ at 56 GPa and ambient temperature.

Atom	x	y	z	U_{eq} (Å^2)
N001	-0.0376(9)	0.4548(7)	0.347(3)	0.0363(19)
N002	0.0423(9)	0.3956(7)	0.412(2)	0.0349(19)
N003	0.0112(8)	0.2798(8)	0.637(3)	0.038(2)
N004	0.0080(9)	0.1611(8)	0.624(3)	0.039(2)
N005	0.1190(9)	0.4401(8)	0.926(3)	0.0363(18)
N006	0.1282(10)	0.3482(7)	0.860(3)	0.0321(16)
N007	0.2446(9)	0.3991(8)	0.624(3)	0.0336(19)
N008	0.2561(8)	0.3186(7)	0.537(3)	0.0334(18)
N009	0.3736(9)	0.3482(7)	0.842(3)	0.0340(18)
N010	0.3790(9)	0.4375(9)	0.925(3)	0.039(2)
N011	0.4970(10)	0.1580(8)	0.630(3)	0.036(2)
N012	0.4891(8)	0.2759(9)	0.637(2)	0.039(2)
N013	0.4627(10)	0.1014(8)	0.897(3)	0.0373(19)
N014	0.5447(10)	0.0423(8)	0.860(3)	0.0352(18)
N015	0.6091(10)	0.3987(7)	0.832(3)	0.0356(19)
N016	0.6332(9)	0.3123(8)	0.901(3)	0.0355(18)
N017	0.7461(12)	0.6025(8)	0.844(3)	0.032(2)
N018	0.7521(12)	0.6257(8)	0.717(3)	0.032(2)
N019	0.6966(10)	0.3992(7)	0.582(3)	0.0346(19)
N020	0.8019(10)	0.3977(8)	0.566(3)	0.039(2)
N021	0.8899(8)	0.3997(7)	0.820(2)	0.0259(16)
N022	0.8693(9)	0.3160(8)	0.895(3)	0.0318(17)
N023	0.7525(13)	0.1369(9)	0.755(3)	0.035(2)
N024	0.7503(12)	0.1407(8)	0.654(3)	0.0275(19)

Supplementary Table 2 | Final Coordinates and Equivalent Isotropic Displacement Parameters for $\iota-N_2$ at 56 GPa.

Atoms	Bond distance (Å)
N001-N002	1.095(13)
N003-N004	1.059(10)
N005-N006	1.00(2)
N007-N008	1.05(3)
N009-N010	1.07(2)
N011-N012	1.050(11)
N013-N014	1.03(2)
N015-N016	0.998(19)
N017-N018	1.13(4)
N019-N020	1.055(18)
N021-N022	1.01(2)
N023-N024	0.88(5)

Supplementary Table 3 | Bond Distances for $\iota-N_2$ at 56 GPa.

References

1. Olijnyk, H. & Jephcoat, A. P. Vibrational Dynamics of Isotopically Dilute Nitrogen to 104 GPa. *Phys. Rev. Lett* **83**, 332–335 (1999).

2. Gregoryanz, E., Goncharov, A. F., Hemley, R. J., Mao, H.-k., Somayazulu, M. & Guoyin S. Raman, infrared, and x-ray evidence for new phases of nitrogen at high pressures and temperatures. *Phys. Rev. B* **66**, 224108 (2002).

3. Pickard, C. J. & Needs, R. J. High-pressure phases of nitrogen. *Phys. Rev. Lett.* **102**, 1–4 (2009).
