# Supplementary material for: Unusually complex phase of dense nitrogen at extreme conditions
Source: Nat Commun. 2018 Nov 9;9:4717. doi: 10.1038/s41467-018-07074-4 (PMC6226474; doi:10.1038/s41467-018-07074-4)
Supplement: Supplementary file 2 — Description of Additional Supplementary Files [file 41467_2018_7074_MOESM2_ESM.docx]

**Description of Additional Supplementary Files**

**File Name: Supplementary Movie 1:**

**Description: The phase transition from ε-N2 to ι-N2 at 750 K and 65 GPa.**
